# Effects of Structured Lipid Supplementation for Eight Weeks on Substrate Utilization During Moderate Intensity Exercise in Healthy Untrained Men

**DOI:** 10.3390/nu18040567

**Published:** 2026-02-09

**Authors:** Chen Wang, Jin-Yu Qi, Li Han, Kaori Yokoi, Kenichi Yanagimoto, Xin-Tang Wang, Zi-Long Fang, Shi-Lun Hou

**Affiliations:** 1 School of Sports Medicine and Rehabilitation, Beijing Sport University, Beijing 100084, China; wangchen_7073@163.com (C.W.);; 2 Nippon Suisan Kaisha, Ltd. (Nissui Corporation), Tokyo 100-8686, Japankaori_yokoi@nissui.co.jp (K.Y.);

**Keywords:** structured lipids, *n*-3 polyunsaturated fatty acids, medium-chain fatty acids, exercise metabolism, endurance performance, erythrocyte fatty acids, healthy untrained men

## Abstract

**Background**: Structured lipids, composed of re-esterified triacylglycerols containing eicosapentaenoic acid (EPA), docosahexaenoic acid (DHA), and medium-chain fatty acids, may influence metabolism and endurance performance. This trial aimed to evaluate the effects of eight weeks of structured lipid supplementation on substrate utilization, erythrocyte fatty acid content, and endurance performance in healthy untrained men. **Methods**: In a double-blind, placebo-controlled, randomized design, 36 participants (18 per group) received either structured lipids or placebo supplementation for eight weeks. Pre- and post-supplementation assessments included maximal oxygen uptake, time to exhaustion, substrate oxidation during exercise at 65% VO_2max_, and erythrocyte membrane fatty acid content. Non-parametric statistical methods were used to analyze within- and between-group differences. **Results**: After supplementation, the structured lipids group showed statistically significant within-group changes in substrate utilization, including lower respiratory exchange ratio and higher percentage fat oxidation, total fat oxidation, and mean fat oxidation rate. Statistically significant increases were also observed in erythrocyte EPA + DHA content and time to exhaustion. Compared with the placebo group, the structured lipids group showed statistically significant post-intervention differences in substrate oxidation, erythrocyte EPA + DHA levels, and time to exhaustion. **Conclusions**: Eight weeks of structured lipid supplementation increased erythrocyte membrane EPA and DHA and enhanced fat oxidation during moderate-intensity exercise in untrained men. Although endurance performance improved, the change was within natural variability and showed substantial interindividual differences. Further rigorously controlled studies are needed to determine whether these metabolic adaptations yield meaningful functional benefits.

## 1. Introduction

Improving endurance performance provides important benefits for cardiovascular function and overall metabolic flexibility, particularly in individuals without regular training [1,2]. Given the abundance of fat reserves, promoting fat oxidation during aerobic exercise is beneficial for enhancing metabolic flexibility [1,3]. Among potential nutritional approaches, supplementation with omega-3 polyunsaturated fatty acids (*n*-3 PUFAs) and medium-chain triglycerides (MCTs) have received increasing attention [4,5].

*n*-3 PUFAs, notably eicosapentaenoic acid (EPA) and docosahexaenoic acid (DHA), are crucial for human health and have been investigated for their potential to modulate exercise metabolism [6,7]. Although some studies have reported improved erythrocyte deformability and oxygen delivery following *n*-3 PUFA intake [8,9], more recent evidence indicates minimal effects in healthy individuals [10], highlighting uncertainty regarding their physiological impact. Similarly, the ergogenic potential of *n*-3 PUFAs remains inconsistent, with reports ranging from enhanced fatty-acid oxidation to null performance effects [6,7,11,12]. MCTs, consisting of medium-chain fatty acids (MCFAs), provide a rapidly oxidizable energy source that can enter mitochondria independent of carnitine transport [13,14], indicating potential to influence metabolism and support endurance performance. Although acute supplementation has yielded inconsistent performance outcomes [15,16], longer-term intake appears to enhance fatty acid oxidation and endurance in some contexts [17,18,19]. These findings suggest that, similar to *n*-3 PUFAs, the ergogenic potential of MCTs is still under debate and may depend on study design, dosage, and participant characteristics [20,21].

A potential explanation for these inconsistencies is the training status of participants. Most studies showing no metabolic or performance improvements were conducted in well-trained athletes [12,15], who may already possess highly developed mitochondrial density, fatty acid oxidation capacity, and cardiovascular adaptations [22]. By contrast, evidence from untrained individuals suggests a greater potential for metabolic and performance benefits [6,23], likely due to their lower baseline efficiency and larger adaptive reserve [2].

Structured lipids (SLs) represent a newer class of functional lipids in which specific fatty acids are re-esterified onto the glycerol backbone, potentially altering their digestion, absorption, and metabolic fate [24,25,26]. This structural modification theoretically enables simultaneous delivery of long-chain *n*-3 PUFAs and rapidly oxidizable MCFAs, offering a mechanistic rationale for enhanced fat utilization during aerobic exercise. However, despite this conceptual advantage, only a few studies have examined SLs enriched with EPA, DHA, and MCFAs, and those reporting improved exercise endurance in untrained individuals did not evaluate whether physiological changes were accompanied by measurable alterations in erythrocyte fatty-acid composition or substrate oxidation [4,5]. Consequently, the mechanisms underlying these functional improvements remain unclear.

Taken together, existing evidence suggests that long-term supplementation with *n*-3 PUFAs, MCTs, or their structured combinations may influence substrate utilization, but findings remain inconsistent and mechanistic data are limited. In particular, it is unknown whether SLs induce measurable changes in erythrocyte fatty-acid profiles or whether such changes are associated with improved endurance tolerance in untrained populations. Accordingly, the present study aimed to investigate the metabolic effects of eight weeks of structured lipid supplementation—specifically substrate utilization and erythrocyte fatty-acid composition—during moderate-intensity aerobic exercise in healthy untrained men. Endurance performance (time to exhaustion) was included as a secondary functional outcome to evaluate whether metabolic adaptations translate into measurable improvements in exercise tolerance.

## 2. Materials and Methods

### 2.1. Experimental Design

A double-blind, placebo-controlled, randomized trial was conducted. The study protocol was approved by the Ethics Committee of Sports Science Experiments of Beijing Sport University (2019065H) and registered on the Chinese Clinical Trial Registry (ChiCTR1900025775). Written informed consent was obtained from all participants prior to enrollment.

A total of 36 participants were enrolled in this study. They were randomly assigned to either the structured lipids group (SG) or the placebo group (PG), with 18 subjects in each group. Randomization considered participants’ age, height, body weight (BW), and body mass index (BMI) to achieve balanced baseline characteristics, as detailed in Table 1. Randomization was performed using a computer-generated permuted block sequence (block size = 4) by an investigator not involved in participant recruitment or data collection. Allocation concealment was ensured by using sequentially numbered, opaque, sealed envelopes prepared by a third party. The study supplements for both groups were identical in appearance, packaging, and labeling. Participants, investigators, and outcome assessors remained blinded to group allocation throughout the entire study period. Blinding was only broken after all data were analyzed. Erythrocyte membrane fatty acid profile, maximal oxygen uptake (VO_2max_), TTE, and substrate oxidation during exercise at 65% VO_2max_ (before supplementation) were assessed at before and after supplementation.

### 2.2. Participants

Inclusion criteria were as follows. Participants were classified as “untrained” based on the absence of any structured or systematic endurance or resistance training for at least the previous six months. They engaged only in normal daily physical activity and did not participate in organized sports or regular exercise programs. (1) Males at an age of 20 years or above, (2) keeping their routine physical activity and dietary pattern without regular exercise training before and during the trial. Exclusion criteria were as follows. (1) Indication of any contraindication to the trial during the initial physical assessment in light of the Physical Activity Readiness Questionnaire; (2) any coronary artery disease risk factor based on the American College of Sports Medicine; (3) any injuries of the muscle and skeleton, or chronic pain of the knee and ankle; and (4) supplementation with *n*-3 PUFAs and/or MCTs as a dietary supplement or otherwise in the last 3 months.

### 2.3. Intervention

The SLs used in this study were provided by Nissui Corporation (Tokyo, Japan). This specific supplement has been used in prior research, which defined it as SLs to distinguish it from a physical mixture. It was synthesized via chemical interesterification of fish oil (as a source of EPA and DHA) and medium-chain triglycerides (MCTs, as a source of C8:0 and C10:0). This process yields a final product mainly composed of re-esterified mixed-chain triglycerides containing both long-chain omega-3 fatty acids and medium-chain fatty acids within individual glycerol molecules [4,5].

Participants ingested the soft gels (0.44 g/each gel) twice daily for 8 weeks, after breakfast (5 gels) and dinner (5 gels). The daily intake in the SG provided 600 mg EPA, 260 mg DHA, and 1730 mg MCFAs, while the PG received corn oil rich in long-chain fatty acids (C18:1 and C18:2 *n*-6) and negligible EPA/DHA. The fatty acid composition of both SLs and corn oil is shown in Appendix A Table A1. The specific daily dosage of 600 mg EPA and 260 mg DHA was chosen to exactly match the efficacious dose of the identical SL supplement reported in prior human trials [4,5]. The MCFA content was consistent with this formulation. Corn oil was selected as the placebo because it is commonly used in lipid supplementation trials and does not contain EPA, DHA, or MCFAs [27]. The placebo capsules were matched to the structured lipid capsules in total energy content, appearance, texture, and flavor to ensure blinding. Participants could not distinguish between supplements based on taste, smell, or capsule color.

Potential adverse events were monitored through spontaneous self-reporting by participants during the study, and no adverse events were reported. All staff and participants were blinded to group allocation until the completion of data collection. Compliance with supplementation was assessed by soft gel counts at follow-up visits and participant self-reports, and adherence was generally high. Participants were also instructed to maintain their habitual physical activity levels throughout the intervention period. No structured training program was introduced, and participants confirmed that no major changes in their daily activity routines occurred during the study.

Although detailed dietary records were not collected, several measures were taken to minimize dietary confounding. Participants received standardized verbal instructions at each visit to avoid changing their habitual diet, particularly sources of dietary fat, and to refrain from consuming any foods or supplements containing EPA, DHA, or MCFAs. Participants were asked to report any substantial dietary change during the study period, and no deviations were noted.

### 2.4. Blood Sampling and Pretreatment

Blood samples were collected from each participant in the morning after a 12 h overnight fast, both immediately before and after the 8-week supplementation period. On each occasion, 2 mL of blood was collected by venipuncture from an antecubital vein into a vacuum tube containing K_2_EDTA as an anticoagulant.

The samples first underwent a 10 min centrifugation (Allegra X-12R, Beckman Coulter, Indianapolis, IN, USA) at 2000× *g* (4 °C) to obtain the erythrocytes. Then, the erythrocytes were washed with physiological saline solution followed by 10 min centrifugation twice at 2000× *g* to obtain the prepared erythrocytes. Finally, erythrocyte membrane fatty acid profile was determined by the Gas Chromatography–Mass Spectroscopy (GC/MS) technique.

### 2.5. Fatty Acid Profile Analysis

The erythrocyte fatty acid derivatization was performed according to the modified Rodrigues method [28]. Firstly, 200 μL of prepared erythrocyte suspension was aliquoted into a 10 mL screw-capped tube. Subsequently, 1.0 mL of 2.5% (*v*/*v*) sulfuric acid/methanol solution was added, followed by vortex mixing for 30 s. The mixture was then heated at 90 °C for 60 min in a water bath. After cooling to room temperature, 1.0 mL of saturated sodium chloride solution and 5 mL of hexane were added sequentially. The solution was vigorously shaken for 10 min and centrifuged at 3000× *g* (6 °C) for 20 min (Allegra X-12R). Finally, 5 μL of the hexane phase was injected into the GC/MS system for analysis.

The analysis was conducted using a QP2010 GC/MS system equipped with an AOC-20 autosampler (Shimadzu, Kyoto, Japan). Separation was achieved on an HP-5 capillary column (25 m × 0.25 mm × 0.25 μm; Agilent, Santa Clara, CA, USA) with the following temperature program. The initial oven temperature was maintained at 60 °C for 1 min, then increased to 220 °C at a rate of 5 °C/min and held for 8 min, followed by a second ramp to 250 °C at 20 °C/min. The total run time was 42.5 min.

Mass spectra were acquired in the range of 40~500 *m*/*z* with a threshold intensity of 1000. For fatty acid identification, a derivatized 37-fatty acid standard mixture (FAMQ-005, AccuStandard, New Haven, CT, USA) was used to establish retention time references. Peak integration and relative quantification (area %) were performed using the ChemStation software (version for QP2010).

### 2.6. Graded Exercise Test (GXT)

Following blood collection, the GXT was performed on an electronically braked cycle ergometer (Monark Ergomedic 839E, Vansbro, Sweden) to determine VO_2max_. The test protocol comprised a 3 min warm-up at 50 W, followed by an exercise phase starting at 100 W. The workload increased by 25 W every 2 min. Participants were required to maintain a pedaling cadence of 60 revolutions per minute throughout the test. Expired gas variables were measured using a gas analyzer system (Cosmed Quark CPET, Albano Laziale, Italy), and heart rate (HR) was continuously recorded. Volitional exhaustion was considered achieved, and the test was terminated, when any three of the following criteria were met [29]:

(1) VO_2_ reached a plateau (defined as an increase of less than 2 mL/kg/min or 150 mL/min) despite an increase in workload; (2) a Borg rating of perceived exertion > 17; (3) a peak respiratory exchange ratio (RER) > 1.10; and (4) HR exceeded 180 beats per minute.

### 2.7. Sustained Exercise Tests (SET)

To minimize procedural learning effects, all participants received standardized instructions and completed a short practice trial on the cycle ergometer before the first graded exercise test. The SET was conducted 3 days after the GXT. For the 48 h period preceding each test, participants were instructed to refrain from consuming alcohol or caffeine and to avoid strenuous exercise. After a 12 h overnight fast, all tests were conducted between 8:00 AM and 12:00 PM.

Participants remained fasted during the test but were allowed water ad libitum. They exercised at a workload corresponding to 65% of their pre-supplementation VO_2max_. This intensity was selected because it represents a moderate, submaximal domain that is commonly used to evaluate endurance tolerance and substrate utilization, while allowing participants to sustain exercise long enough for meaningful metabolic measurements [30,31]. The target workload was calculated individually from the linear regression between VO_2_ and power output obtained during the pre-supplementation graded exercise test. The endurance test was terminated when participants reached volitional exhaustion or when any of the following predefined criteria were met: (1) inability to maintain the required cadence (±5 rpm) for more than 10 s despite verbal encouragement; or (2) onset of signs or symptoms of exercise intolerance, including chest pain, severe dyspnea, dizziness, or nausea. Time to exhaustion (TTE) was recorded as the total duration for which the participant was able to maintain the prescribed workload.

Expired gas samples were collected using the Quark CPET system for 5 min at rest, for 5 min at 20 min intervals during exercise, and at the end of exercise. Substrate oxidation rates during the exercise test were estimated using non-protein respiratory quotient equations [32]:

CHO oxidation (CHOO) rate (g/min) = 4.585 × VCO_2_ (L/min) − 3.226 × VO_2_ (L/min)

Fatty acid oxidation (FAO) rate (g/min) = 1.695 × VO_2_ (L/min) − 1.701 × VCO_2_ (L/min)

For each subject, the total CHOO (g) and FAO (g) were evaluated with the area under the oxidation rate (g/min) − time (min) curve, the total energy expenditure (EE) (kcal) equaled total CHOO (g) × 4 + total FAO (g) × 9, the mean CHOO rate (g/min) was calculated from total CHOO (g) ÷ time (min), the mean FAO rate (g/min) was calculated from total FAO (g) ÷ time (min), the mean EE rate (kcal/min) was calculated from total EE (kcal) ÷ time (min), the FAO percentage (%) equaled total FAO (g) × 9 ÷ total EE (kcal) × 100, and the mean RER was evaluated from the area under the RER–time curve, equaling the area ÷ time (min).

### 2.8. Statistical Analysis

Data were analyzed using SPSS version 26.0 (IBM Corp., Armonk, NY, USA). The normality of continuous variables was assessed using the Shapiro–Wilk test, while the homogeneity of variances was tested using Levene’s test. As most outcome variables did not satisfy the assumptions of normality or homogeneity, non-parametric methods were applied for the primary analyses. Within-group comparisons (before vs. after intervention) were performed using the Wilcoxon signed-rank test, and between-group comparisons (SG vs. PG) were conducted using the Mann–Whitney U test. Effect sizes for non-parametric comparisons were reported using r, calculated as $r=\frac{z}{\sqrt{n}}$, where z is the test statistic from the Wilcoxon test and *n* is the total number of observations. This measure provides an estimate of the magnitude of the difference independent of sample size and is commonly used as an effect-size index for non-parametric analyses.

Baseline characteristics (age, height, body weight, and BMI) are presented as mean ± standard deviation, while all other variables are expressed as median (interquartile range). All *p*-values were two-tailed, with statistical significance set at *p* < 0.05. Given the small sample size and the exploratory nature of this study, no formal adjustment for multiple comparisons was performed.

## 3. Results

At baseline, there were no significant differences between the SG and the PG in substrate oxidation parameters, erythrocyte fatty acid content, VO_2max_ and TTE.

### 3.1. Substrate Oxidation at the Workload Equivalent to 65% VO_2max_

**Figure 1:**
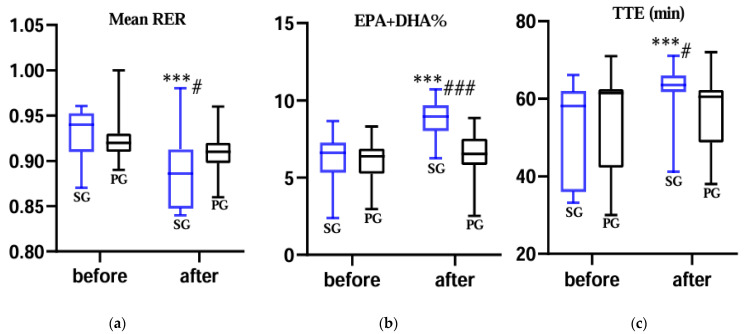
(**a**) Changes in mean RER during exercise to exhaustion at 65% VO_2max_ before and after supplementation. (**b**) Changes in the combined EPA + DHA content (%) in the erythrocyte membrane before and after supplementation. (**c**) Changes in TTE during exercise at 65% VO_2max_ before and after supplementation. SG: structured lipids group; PG: placebo group; TTE: time to exhaustion; RER: respiratory exchange ratio; EPA: eicosapentaenoic acid; DHA: docosahexaenoic acid. ***: *p* < 0.001 compared to pre-supplementation within the same group. #: *p* < 0.05, ###: *p* < 0.001 compared to the post-supplementation value of the SG vs. PG. No adjustment was made for multiple comparisons; reported *p*-values are uncorrected.

At 65% VO_2max_, SL supplementation significantly enhanced fat utilization in the SG. RER decreased (median from 0.94 to 0.88 in the SG, *p* < 0.001), while no significant change was observed in the PG. After supplementation, SG showed significantly lower RER compared to PG (median 0.88 vs. 0.91, *p* = 0.013), indicating increased fat oxidation (Figure 1a).

In SG, fat oxidation percentage increased significantly (median from 13.46% to 31.22%, *p* < 0.001), with total fat oxidation rising (median from 6.93 g to 20.65 g *p* < 0.001) and mean fat oxidation rate increasing (median from 0.14 g/min to 0.34 g/min, *p* < 0.001). In PG, modest increases were observed (median FAO%: from 17.38% to 21.14%, *p* = 0.014; total FAO: from 9.59 g to 15.52 g, *p* = 0.031; mean FAO rate: from 0.18 g/min to 0.23 g/min, *p* = 0.013). After supplementation, SG had significantly higher fat oxidation compared to PG (*p* < 0.05 for all). In contrast, mean CHO oxidation decreased in both groups (SG: from 2.01 g/min to 1.63 g/min, *p* < 0.001; PG: from 2.06 g/min to 1.80 g/min, *p* = 0.021), with no significant group difference post-supplementation. Detailed data for all variables, including four quartile ranges, are provided in Table 2 and Appendix A Table A2.

### 3.2. Erythrocyte Membrane Fatty Acid Profile

After supplementation, the SG showed significant increases in erythrocyte EPA (median from 0.86% to 2.32%, *p* < 0.001), DHA (median from 5.48% to 6.61%, *p* = 0.004), and EPA + DHA (median from 6.56% to 8.89%, *p* < 0.001), while no significant change was observed in the PG (Figure 1b). After intervention, the SG also had significantly higher levels than the PG in EPA (median 2.32% vs. 1.19%, *p* < 0.001), DHA (median 6.61% vs. 5.41%, *p* < 0.001), and EPA + DHA (median 8.89% vs. 6.54%, *p* < 0.001). The *n*-3/*n*-6 PUFA ratio similarly increased within the SG and was higher than in the PG post-supplementation. Detailed data for all variables are presented in Table 3 and Appendix A Table A3.

### 3.3. VO_2max_ and TTE at the Workload Equivalent to 65% VO_2max_

VO_2max_ showed no significant change in either the SG or the PG after supplementation.

TTE increased significantly in SG after supplementation (median 58.00 min to 63.50 min, *p* < 0.001), whereas no significant change was observed in PG. After supplementation, TTE was significantly higher in SG compared with PG (median 63.50 min vs. 60.50 min, *p* = 0.013) (Figure 1c). Detailed data for all variables are presented in Table 2.

## 4. Discussion

The present study examined the effects of eight weeks of SL supplementation on substrate utilization, erythrocyte fatty acid composition, and endurance performance in untrained men. Overall, SL supplementation increased fat oxidation during moderate-intensity exercise and elevated erythrocyte omega-3 polyunsaturated fatty acids (EPA and DHA), accompanied by prolongation of exercise time.

The observed increase in fat oxidation during moderate-intensity exercise is consistent with previous findings, which report a greater reliance on fatty acid utilization when using similar structured lipid formulations [4]. These trends may result from a potential synergistic effect between *n*-3 PUFAs and MCFAs within SLs. While both *n*-3 PUFAs and MCTs have been shown to independently promote fatty acid oxidation, the exact mechanisms through which they interact in SLs remain speculative. Previous studies have demonstrated that EPA and DHA supplementation can enhance fatty acid oxidation during endurance exercise in both older women and young untrained men [6,23,33], while continuous MCT intake has also been associated with increased fatty acid oxidation and improved endurance performance [18,34]. In SLs, MCFAs might serve as more readily available substrates [13,14] that could potentially interact with *n*-3 PUFAs to support metabolic adaptation. However, these proposed interactions were not directly examined in this study and remain a hypothesis. Moreover, given the lack of plasma MCFA measurements, it is unclear whether the observed metabolic shifts reflect immediate substrate availability, longer-term adaptations, or a combination of both.

Another notable finding was that the observed elevation in erythrocyte EPA and DHA levels following SL supplementation aligns with previous research demonstrating membrane incorporation of *n*-3 polyunsaturated fatty acids within several weeks of intake [21,35,36]. Such incorporation may modify erythrocyte deformability and oxygen transport [27,33], and consequently facilitate greater fat oxidation via alterations in membrane composition and mitochondrial signaling pathways involving PGC-1α [37,38]. However, these potential mechanisms were not directly assessed in this study, and thus remain hypothetical rather than confirmatory.

Although the SG showed a within-group increase in time to exhaustion (median 58.0 to 63.5 min, *p* < 0.001; r = 0.83), this finding should be interpreted cautiously. Baseline TTE values were slightly lower in the SG than in the PG, although not significantly so (*p* = 0.409; r = 0.13), and the magnitude of improvement was comparable to the natural variability commonly observed in untrained individuals. After 8 weeks, the between-group difference reached statistical significance (*p* = 0.013; r = 0.41), but the effect size was moderate and the analysis did not include adjustment for multiple comparisons. In addition, TTE was a secondary exploratory outcome, and several alternative explanations must be considered, including day-to-day fluctuations, increased familiarity with the testing protocol, placebo effects, and regression to the mean. Collectively, these factors indicate that the observed TTE changes represent preliminary signals rather than definitive evidence of an ergogenic effect.

The changes observed in metabolic parameters in our untrained cohort contrast with the inconsistent outcomes reported in well-trained athletes [39,40,41]. One possible explanation could relate to differences in adaptive reserve, as highly trained individuals already possess optimized mitochondrial density, fatty acid oxidation capacity, and cardiovascular adaptations. Untrained subjects, on the other hand, start from lower baseline efficiency and may therefore exhibit greater potential for metabolic improvements [2,22]. Although the supplementation regimen in this study used a relatively lower daily dose [18,23], the longer intervention period may have contributed to preliminary metabolic trends.

This study specifically targeted healthy untrained men and incorporated erythrocyte fatty-acid profiling to provide preliminary mechanistic insight into the effects of SLs on substrate utilization. Nevertheless, several limitations must be acknowledged. Gastrointestinal symptoms associated with MCFAs were monitored only through spontaneous self-reporting, which may underestimate their true incidence. Detailed dietary monitoring and assessment of additional supplement use were not conducted, and although participants were instructed to maintain habitual dietary patterns, the absence of food records precludes ruling out unmeasured variations in dietary fat intake as potential confounders. Physical activity during the intervention was also not objectively monitored, and natural fluctuations in daily activity may have influenced metabolic or performance outcomes. The exclusive inclusion of men limits generalizability, and the relatively small sample size reduces statistical power to detect modest effects. Although non-parametric analyses were adopted to accommodate the distributional characteristics of the data, multiple outcomes were evaluated without formal adjustment for multiplicity, increasing the likelihood of type I error. In addition, an a priori sample size calculation or power analysis was not conducted, which should be acknowledged as an additional methodological limitation. Finally, although the study received financial support from Nissui Corporation, all experimental procedures, data analyses, and interpretations were conducted independently; however, the possibility of subtle sponsor-related bias cannot be entirely excluded. Taken together, these limitations highlight the preliminary nature of the findings and underscore the need for larger, rigorously controlled randomized trials.

## 5. Conclusions

In conclusion, eight weeks of structured lipid supplementation led to measurable increases in erythrocyte membrane EPA and DHA content and was accompanied by increased fat oxidation during moderate-intensity exercise in untrained men. Although time-to-exhaustion increased in the supplementation group, the magnitude of change was comparable to natural variability and accompanied by considerable interindividual differences, indicating that this finding should be interpreted as a preliminary signal rather than conclusive evidence of an ergogenic effect. Larger, rigorously controlled trials with objective monitoring of diet, physical activity, and mechanistic biomarkers are needed to determine whether the observed metabolic adaptations translate into meaningful functional benefits.

## Figures and Tables

**Table 1:** Physical characteristics of the subjects.

Physical Characteristics	PG (*n* = 18)	SG (*n* = 18)
Age (yr)	21.3 ± 1.9	21.3 ± 2.1
Height (cm)	178.3 ± 4.3	178.9 ± 7.1
Body weight (kg)	76.0 ± 8.6	75.5 ± 10.7
BMI (kg/m^2^)	23.9 ± 2.5	23.6 ± 2.9

PG: placebo group; SG: structured lipids group; BMI: body mass index.

**Table 2:** Changes in endurance performance and substrate oxidation before and after supplementation.

Variable	Group	Before Median (IQR)	After Median (IQR)	*p*, r and 95%CI Within SG	*p*, r and 95%CI Within PG	*p*, r and 95%CI Between Before	*p* and r (95%CI) Between After
Mean RER	SG	0.94 (0.91–0.95)	0.88 (0.84–0.91)	*p* < 0.001 r = 0.78 95%CI: −0.05 (−0.06, −0.02)	*p* = 0.080 r = 0.41 95%CI: −0.01 (−0.02, 0.00)	*p* = 0.237 r = 0.19 95%CI: 0.01 (−0.01, 0.03)	*p* = 0.013 r = 0.41 95%CI: −0.03 (−0.05, −0.01)
	PG	0.92 (0.91–0.93)	0.91 (0.89–0.92)				
FAO (%)	SG	13.46 (8.84–19.39)	31.22 (22.69–40.11)	*p* < 0.001 r = 0.81 95%CI: 15.37 (10.20, 21.57)	*p* = 0.014 r = 0.58 95%CI: 5.80 (1.51, 9.92)	*p* = 0.376 r = 0.14 95%CI: −2.12 (−6.35, 3.79)	*p* = 0.013 r = 0.41 95%CI: 10.06 (2.69, 16.88)
	PG	17.38 (11.85–20.33)	21.14 (18.90–27.68)				
Total FAO (g)	SG	6.93 (5.35–11.09)	20.65 (16.22–24.48)	*p* < 0.001 r = 0.85 95%CI: 10.49 (6.93, 14.24)	*p* = 0.031 r = 0.50 95%CI: 2.89 (0.39, 6.01)	*p* = 0.319 r = 0.16 95%CI: −1.99 (−5.00, 1.74)	*p* < 0.001 r = 0.54 95%CI: 7.41 (3.58, 10.50)
	PG	9.59 (6.72–12.69)	15.52 (9.55–16.39)				
Mean FAO rate (g/min)	SG	0.14 (0.09–0.19)	0.34 (0.25–0.39)	*p* < 0.001 r = 0.81 95%CI: 0.15 (0.09, 0.21)	*p* = 0.013 r = 0.58 95%CI: 0.05 (0.01, 0.10)	*p* = 0.310 r = 0.16 95%CI: −0.02 (−0.07, 0.03)	*p* = 0.009 r = 0.43 95%CI: 0.10 (0.03, 0.15)
	PG	0.18 (0.12–0.22)	0.23 (0.19–0.26)				
VO_2max_ (ml/min/kg)	SG	38.85 (33.27–42.97)	37.33 (32.55–42.80)	*p* = 0.231 r = 0.28 95%CI: −0.94 (−2.39, 0.66)	*p* = 0.184 r = 0.31 95%CI: −1.27 (−3.78, 0.80)	*p* = 0.635 r = 0.07 95%CI: −1.05 (−4.70, 3.10)	*p* = 1.000 r < 0.001 95%CI: 0.07 (−3.90, 4.07)
	PG	38.20 (36.15–44.32)	36.89 (32.93–42.80)				
TTE (min)	SG	58.00 (36.00–62.00)	63.50 (61.75–66.00)	*p* < 0.001 r = 0.83 95%CI: 8.50 (4.00, 15.50)	*p* = 0.887 r = 0.03 95%CI: 0.00 (−2.50, 9.00)	*p* = 0.409 r = 0.13 95%CI: −1.50 (−9.00, 5.00)	*p* = 0.013 r = 0.41 95%CI: 4.00 (1.00, 11.00)
	PG	61.50 (42.25–62.50)	60.50 (48.75–62.25)				

PG: placebo group; SG: structured lipids group; FAO: fatty acid oxidation; RER: respiratory exchange ratio. Data are presented as median (interquartile range). Within-group differences (pre vs. post) were analyzed using the Wilcoxon signed-rank test, and between-group differences (SG vs. PG) were analyzed using the Mann–Whitney U test. Significant differences: within-group, *p* < 0.05; between-group, *p* < 0.05. Effect sizes are reported as r, representing the magnitude of the observed difference. No adjustment was made for multiple comparisons; reported *p*-values are uncorrected.

**Table 3:** Changes in erythrocyte membrane fatty acid % before and after supplementation.

Variable	Group	Before Median (IQR)	After Median (IQR)	*p*, r and 95%CI Within SG	*p*, r and 95%CI Within PG	*p*, r and 95%CI Between Before	*p* and r (95%CI) Between After
20:5 *n*-3 (EPA)	SG	0.86 (0.61–1.45)	2.32 (1.89–2.58)	*p* < 0.001 r = 0.81 95%CI: 1.29 (0.75, 1.76)	*p* = 0.286 r = 0.25 95%CI: 0.23 (−0.11, 0.52)	*p* = 0.635 r = 0.07 95%CI: 0.07 (−0.24, 0.42)	*p* < 0.001 r = 0.76 95%CI: 1.12 (0.77, 1.45)
	PG	0.80 (0.57–1.37)	1.19 (0.82–1.57)				
22:6 *n*-3 (DHA)	SG	5.48 (3.77–6.40)	6.61 (6.08–7.08)	*p* = 0.004 r = 0.67 95%CI: 1.09 (0.37, 2.03)	*p* = 0.286 r = 0.25 95%CI: 0.25 (−0.29, 1.08)	*p* = 0.311 r = 0.16 95%CI: 0.41 (−0.48, 1.21)	*p* < 0.001 r = 0.56 95%CI: 1.17 (0.58, 1.73)
	PG	5.22 (4.17–5.86)	5.41 (4.79–6.05)				
EPA + DHA	SG	6.56 (5.32–7.24)	8.89 (8.02–9.67)	*p* < 0.001 r = 0.84 95%CI: 2.40 (1.55, 3.36)	*p* = 0.145 r = 0.34 95%CI: 0.68 (−0.28, 1.45)	*p* = 0.393 r = 0.14 95%CI: 0.30 (−0.53, 1.19)	*p* < 0.001 r = 0.67 95%CI: 2.27 (1.43, 3.15)
	PG	6.38 (5.26–6.86)	6.54 (5.82–7.52)				
PUFAs	SG	35.13 (29.70–36.90)	37.35 (35.98–38.03)	*p* = 0.020 r = 0.55 95%CI: 2.75 (0.48, 6.09)	*p* = 0.231 r = 0.28 95%CI: 1.26 (−0.77, 4.88)	*p* = 0.825 r = 0.03 95%CI: −0.23 (−2.68, 2.48)	*p* = 0.359 r = 0.15 95%CI: 0.62 (−0.63, 2.59)
	PG	35.61 (29.26–38.03)	36.58 (34.51–38.02)				
*n*-3/*n*-6 PUFAs	SG	0.54 (0.47–0.67)	0.81 (0.72–0.87)	*p* = 0.002 r = 0.73 95%CI: 0.23 (0.14, 0.28)	*p* = 0.286 r = 0.25 95%CI: 0.04 (−0.03, 0.13)	*p* = 0.924 r = 0.01 95%CI: −0.01 (−0.12, 0.12)	*p* < 0.001 r = 0.64 95%CI: 0.18 (0.11, 0.26)
	PG	0.59 (0.44–0.70)	0.60 (0.55–0.68)				

PG: placebo group; SG: structured lipids group; EPA: eicosapentaenoic acid; DHA: docosahexaenoic acid; PUFAs: polyunsaturated fatty acids. Values are expressed as median (interquartile range) and showed only mean weight percentage of total fatty acids > 1%. Within-group differences (Pre vs. Post) were determined by the Wilcoxon signed-rank test, and between-group comparisons (SG vs. PG) were evaluated using the Mann–Whitney U test. Significant differences: within-group, *p* < 0.05; between-group, *p* < 0.05. Effect sizes are reported as r, representing the magnitude of the observed difference. No adjustment was made for multiple comparisons; reported *p*-values are uncorrected.

## Data Availability

The original contributions presented in this study are included in the article. Further inquiries can be directed to the corresponding authors.

## Abbreviations

BMI	body mass index
CHO	carbohydrate
CHOO	carbohydrate oxidation
DHA	docosahexaenoic acid
EPA	eicosapentaenoic acid
EE	energy expenditure
FAO	fatty acid oxidation
FFA	free fatty acid
GXT	graded exercise test
HR	heart rate
LCTs	long-chain triglycerides
MCFAs	medium-chain fatty acids
*n*-3 PUFAs	omega-3 polyunsaturated fatty acids
PG	placebo group
RER	respiratory exchange ratio
SET	sustained exercise tests
SG	structured lipids group
SLs	structured lipids
TTE	time to exhaustion
VO_2max_	maximal oxygen uptake

## Appendix A

**Table A1:** Fatty acid composition of corn oil and structured lipids.

Fatty Acid	Corn Oil %	Structured Lipids %
8:0 (MCFAs)	0.4	25.3
10:0 (MCFAs)	0.3	19.1
12:0	ND	0.2
14:0	ND	3.2
15:0	ND	0.2
16:0	11.5	3.7
16:1	0.1	5.5
16:2	ND	1.1
16:4	ND	2.4
17:1	ND	0.1
18:0	1.7	0.3
18:1 (LCFA)	29.6	4.9
18:2 *n*-6 (LCFA)	54.2	0.7
18:3 *n*-6	ND	0.2
18:3 *n*-3	1.1	0.5
18:4 *n*-3	ND	2.3
20:0	0.4	ND
20:1	0.3	0.3
20:3 *n*-6	ND	0.1
20:4 *n*-6	ND	0.9
20:4 *n*-3	ND	0.6
20:5 *n*-3 (EPA)	ND	16.0
21:5 *n*-3	ND	0.6
22:0	0.1	ND
22:1	ND	0.2
22:5 *n*-6	ND	0.2
22:5 *n*-3	ND	1.5
22:6 *n*-3 (DHA)	ND	7.0
24:0	0.2	ND

MCFAs: medium-chain fatty acids; LCFA: long-chain fatty acid; EPA: eicosapentaenoic acid; DHA: docosahexaenoic acid; and ND: not detected. Data are presented as a weight percentage of total fatty acids.

**Table A2:** Changes in substrate oxidation before and after supplementation.

Variable	Group	Before Median (IQR)	After Median (IQR)	*p*, r and 95%CI Within SG	*p*, r and 95%CI Within PG	*p*, r and 95%CI Between Before	*p* and r (95%CI) Between After
Total CHOO (g)	SG	105.10 (76.28–142.01)	103.86 (83.43–116.58)	*p* = 0.420 r = 0.19 95%CI: −6.30 (−22.01, 8.49)	*p* = 0.267 r = 0.26 95%CI: −7.89 (−21.29, 11.65)	*p* = 0.849 r = 0.03 95%CI: −1.85 (−28.98, 19.52)	*p* = 0.704 r = 0.06 95%CI: −2.98 (−21.50, 13.28)
	PG	113.79 (77.69–135.29)	104.76 (87.32–119.15)				
Mean CHOO rate (g/min)	SG	2.01 (1.79–2.26)	1.63 (1.33–1.80)	*p* < 0.001 r = 0.87 95%CI: −0.39 (−0.54, −0.25)	*p* = 0.021 r = 0.54 95%CI: −0.17 (−0.30, −0.02)	*p* = 0.975 r = 0.01 95%CI: 0.00 (−0.22, 0.23)	*p* = 0.055 r = 0.31 95%CI: −0.23 (−0.45, 0.00)
	PG	2.06 (1.758–2.23)	1.80 (1.61–2.01)				
Total EE (kcal)	SG	526.54 (363.34–625.49)	572.43 (531.31–655.38)	*p* = 0.028 r = 0.51 95%CI: 63.81 (4.30, 135.45)	*p* = 0.472 r = 0.17 95%CI: −21.12 (−74.56, 100.87)	*p* = 0.728 r = 0.05 95%CI: −16.25 (−130.84, 91.07)	*p* = 0.195 r = 0.21 95%CI: 45.60 (−16.56, 111.65)
	PG	549.35 (370.21–635.87)	547.76 (444.90–581.10)				
Mean EE rate (kcal/min)	SG	9.56 (8.41–10.38)	9.18 (8.60–9.96)	*p* = 0.472 r = 0.16 95%CI: −0.19 (−0.77, 0.38)	*p* = 0.601 r = 0.12 95%CI: −0.14 (−0.77, 0.54)	*p* = 0.862 r = 0.02 95%CI: −0.05 (−7.80, 0.84)	*p* = 0.681 r = 0.06 95%CI: −0.13 (−0.90, 0.49)
	PG	9.83 (8.25–10.47)	9.18 (8.80–10.10)				

PG: placebo group; SG: structured lipids group; CHOO: carbohydrate oxidation; and EE: energy expenditure. Data are presented as median (interquartile range). Within-group differences (pre vs. post) were analyzed using the Wilcoxon signed-rank test, and between-group differences (SG vs. PG) were analyzed using the Mann–Whitney U test. Significant differences: within-group, *p* < 0.05; betwee*n*-group, *p* < 0.05. Effect sizes are reported as r, representing the magnitude of the observed difference. No adjustment was made for multiple comparisons; reported *p*-values are uncorrected.

**Table A3:** Changes in erythrocyte membrane fatty acid % before and after supplementation.

Variable	Group	Before Median (IQR)	After Median (IQR)	*p*, r and 95%CI Within SG	*p*, r and 95%CI Within PG	*p*, r and 95%CI Between Before	*p* and r (95%CI) Between After
16:0	SG	26.92 (25.42–28.95)	25.78 (24.71–26.26)	*p* = 0.011 r = 0.60 95%CI: −1.60 (−3.12, −0.49)	*p* = 0.039 r = 0.48 95%CI: −1.46 (−3.00, −0.12)	*p* = 0.681 r = 0.06 95%CI: −0.31 (−1.93, 1.35)	*p* = 0.849 r = 0.03 95%CI: −0.10 (−1.50, 0.77)
	PG	27.56 (26.16–28.84)	25.50 (24.72–27.28)				
18:2 *n*-9	SG	11.41 (10.46–13.05)	13.34 (12.24–13.93)	*p* = 0.014 r = 0.58 95%CI: 1.67 (0.55, 2.58)	*p* = 0.012 r = 0.59 95%CI: 1.48 (0.35, 2.67)	*p* = 0.635 r = 0.07 95%CI: −0.29 (−2.08, 1.16)	*p* = 0.137 r = 0.24 95%CI: −0.91 (−1.86, 0.21)
	PG	11.79 (10.07–14.11)	14.09 (12.73–15.31)				
18:1 *n*-9	SG	13.17 (12.33–13.60)	12.90 (12.11–13.45)	*p* = 0.420 r = 0.19 95%CI: −0.12 (−0.50, 0.31)	*p* = 0.811 r = 0.56 95%CI: 0.03 (−0.45, 0.47)	*p* = 0.635 r = 0.07 95%CI: −0.18 (−0.86, 0.52)	*p* = 0.071 r = 0.30 95%CI: −0.60 (−1.23, 0.06)
	PG	13.21 (12.43–13.88)	13.55 (12.31–13.86)				
18:0	SG	21.05 (18.88–24.07)	20.21 (19.63–20.78)	*p* = 0.064 r = 0.43 95%CI: −1.25 (−2.73, 0.05)	*p* = 0.420 r = 0.19 95%CI: −0.59 (−2.18, 0.86)	*p* = 0.591 r = 0.09 95%CI: 0.49 (−1.31, 2.25)	*p* = 0.527 r = 0.10 95%CI: 0.19 (−0.50, 0.79)
	PG	20.33 (19.38–22.99)	19.76 (19.36–21.19)				
20:4 *n*-6	SG	14.01 (9.98–14.45)	11.93 (11.13–12.45)	*p* = 0.306 r = 0.24 95%CI: −0.98 (−2.39, 0.82)	*p* = 0.472 r = 0.16 95%CI: −0.51 (−1.61, 1.22)	*p* = 0.591 r = 0.09 95%CI: 0.50 (−1.85, 2.47)	*p* = 0.282 r = 0.17 95%CI: −0.57 (−1.48, 0.41)
	PG	12.15 (10.28–15.14)	12.63 (11.11–13.31)				
20:3 *n*-6	SG	1.15 (0.92–1.29)	1.13 (1.02–1.38)	*p* = 0.064 r = 0.43 95%CI: 0.10 (−0.01, 0.24)	*p* = 0.557 r = 0.13 95%CI: 0.03 (−0.17, 0.17)	*p* = 0.591 r = 0.09 95%CI: 0.04 (−0.15, 0.21)	*p* = 0.242 r = 0.19 95%CI: 0.09 (−0.06, 0.34)
	PG	1.07 (0.94–1.22)	1.13 (0.91–1.22)				
22:3 *n*-3	SG	1.61 (0.75–2.09)	1.51 (1.25–1.82)	*p* = 0.948	*p* = 0.528 r = 0.14 95%CI: 0.13 (−0.35, 0.67)	*p* = 0.899 r = 0.21 95%CI: −0.07 (−0.73, 0.67)	*p* = 0.975 r = 0.010 95%CI: −0.01 (−0.33, 0.31)
	PG	1.42 (0.44–2.44)	1.59 (1.22–2.14)	95%CI: −0.02 (−0.33, 0.66)			

PG: placebo group; SG: structured lipids group. Values are expressed as median (interquartile range) and showed only mean weight percentage of total fatty acids > 1%. Within-group differences (pre vs. post) were determined by the Wilcoxon signed-rank test, and between-group comparisons (SG vs. PG) were evaluated using the Mann–Whitney U test. Significant differences: within-group, *p* < 0.05; betwee*n*-group, *p* < 0.05. Effect sizes are reported as r, representing the magnitude of the observed difference. No adjustment was made for multiple comparisons; reported *p*-values are uncorrected.
